# Does Birth Interval Matter in Under-Five Mortality? Evidence from Demographic and Health Surveys from Eight Countries in West Africa

**DOI:** 10.1155/2021/5516257

**Published:** 2021-05-15

**Authors:** Eugene Budu, Bright Opoku Ahinkorah, Edward Kwabena Ameyaw, Abdul-Aziz Seidu, Betregiorgis Zegeye, Sanni Yaya

**Affiliations:** ^1^Department of Population and Health, University of Cape Coast, Cape Coast, Ghana; ^2^School of Public Health, Faculty of Health, University of Technology Sydney, Ultimo, Australia; ^3^College of Public Health, Medical and Veterinary Sciences, James Cook University, Townsville, Queensland, Australia; ^4^HaSET Maternal and Child Health Research Program, Shewarobit Field Office, Shewa Robit, Ethiopia; ^5^Faculty of Medicine, University of Parakou, Parakou, Benin

## Abstract

In sub-Saharan Africa (SSA), every 1 in 12 children under five dies every year compared with 1 in 147 children in the high-income regions. Studies have shown an association between birth intervals and pregnancy outcomes such as low birth weight, preterm birth, and intrauterine growth restriction. In this study, we examined the association between birth interval and under-five mortality in eight countries in West Africa. A secondary analysis of the Demographic and Health Survey (DHS) data from eight West African countries was carried out. The sample size for this study comprised 52,877 childbearing women (15-49 years). A bivariate logistic regression analysis was carried out and the results were presented as crude odds ratio (cOR) and adjusted odds ratios (aOR) at 95% confidence interval (CI). Birth interval had a statistically significant independent association with under-five mortality, with children born to mothers who had >2 years birth interval less likely to die before their fifth birthday compared to mothers with ≤2 years birth interval [cOR = 0.56; CI = 0.51 − 0.62], and this persisted after controlling for the covariates [aOR = 0.55; CI = 0.50 − 0.61]. The country-specific results showed that children born to mothers who had >2 years birth interval were less likely to die before the age of five compared to mothers with ≤2 years birth interval in all the eight countries. In terms of the covariates, wealth quintile, mother's age, mother's age at first birth, partner's age, employment status, current pregnancy intention, sex of child, size of child at birth, birth order, type of birth, and contraceptive use also had associations with under-five mortality. We conclude that shorter birth intervals are associated with higher under-five mortality. Other maternal and child characteristics also have associations with under-five mortality. Reproductive health interventions aimed at reducing under-five mortality should focus on lengthening birth intervals. Such interventions should be implemented, taking into consideration the characteristics of women and their children.

## 1. Introduction

Under-five mortality is a global indicator of the overall development children's health, and nations' socioeconomic and environmental conditions for both children, household, and community [1]. To deal with this, the United Nations in 2000 signed the Millennial Development Goals, which included the reduction of child mortality and improving maternal health by 2015 [2]. Western Africa is known to have the world's highest MMR rates and one of the highest rates for under-five mortality in children, including at the neonatal stage [3]. Although under-five mortality has reduced significantly, it remains a major health challenge in both low- and middle-income countries (LMICs). SSA, which is the home of most LMICs, has in the last two decades witnessed a decline in under-five mortality. However, it is still the home of persistently high rates of under-five mortality compared with high-income countries (HICs) [4, 5]. This has led to the target 2 of Sustainable Development Goal (SDG) 3 which aims to reduce under-five mortality to at least 25 per 1000 live births by 2030 [6].

In SSA, every 1 in 12 children under five years dies every year compared with 1 in 147 children in the HICs [7]. The high under-five mortality in SSA can be avoided with improved coverage of immunization, prevention, early treatment, and control of malaria and diarrhea as well as other prevalent childhood diseases [8]. Notable measures have been put in place to reduce under-five mortality through strengthening healthcare systems, increasing the supply of primary healthcare facilities and services to the local community [9].

However, literature has shown that in LMICs, education, household size, and age of women are essential determinants of under-five mortality [10]. For instance, education has been found to improve the knowledge and skills of the mother to effectively access, understand, and utilize information and resources which are helpful for child health and overall development [11]. Large family size can also expose children to a high risk of death due to constraints on family finances, while early and late maternal age has been found to increase the risk of under-five mortality due to less use of maternal and child healthcare services attributed to the barriers to healthcare access [11]. Apart from these factors, studies have also shown that the time between a previous live birth and index live birth may have adverse outcomes including under-five mortality, low birth weight, preterm birth, and intrauterine growth restriction [12]. This is because a woman may not have physiologically recovered from the previous birth if she conceives the next child in less than 36 months [12]. Birth interval, therefore, plays a major role in child mortality and the World Health Organization recommends that women space their births at least three years and at most five years to reduce maternal and child health risks [13]. Short birth spacing whether intended or otherwise will have negative outcomes on the physiologic and anatomic capacity of the woman [12]. This is because intervals less than three years could be associated with an elevated risk of infant death or injury [14]. In West Africa, under-five mortality rates are more than twice the rate for most countries in other parts of the continent [13]. However, studies on under-five mortality in West Africa have focused so much attention on the demographic and socioeconomic factors associated with under-five mortality. There is therefore a dearth of evidence on the association between birth interval and under-five mortality in West Africa [15–18]. Hence, this study examined the association between birth interval and under-five mortality in eight countries in West Africa.

## 2. Methods and Materials

### 2.1. Study Design and Data Source

A secondary analysis of the Demographic and Health Survey (DHS) data on women in West Africa was done from eight West African countries in Burkina Faso (2010), Benin (2018-2018), Cote D'Ivorie (2011-2012), Gambia (2013), Mali (2018), Nigeria (2018), Sierra Leone (2019), and Togo (2013-2014). The DHS is a nationally representative survey designed to collect data on various health topics such as nutrition, mortality, domestic violence, and female genital mutilation, access to mass media, fertility, young child development, breastfeeding and food intake, vaccinations, and treatment of diseases. A stratified dual-stage sampling approach was employed, and the same questions were posed to women of all these countries and thus make it feasible for multicountry study. The study involved a multistage sampling process (i.e., enumeration areas [EAs]), followed by systematic household sampling within the selected EAs. The sample size for this study comprised 52,877 childbearing women (see Table 1) between the ages of 15-49. We followed the “Strengthening the Reporting of Observational Studies in Epidemiology” (STROBE) guidelines in conducting this study. The data set is freely available to the public at https://dhsprogram.com/data/available-datasets.cfm.

### 2.2. Definition of Variables

#### 2.2.1. Outcome Variables

The study used under-five mortality as the outcome variable. Under-five mortality, also known as child mortality, refers to the death of infants and children under the age of five. This variable took a binary form; such that under-five mortality will be regarded as a success (1 = if death occurs in the specified age period) or failure (0 = if the child is alive in the specified age period).

#### 2.2.2. Independent Variable

The study used birth interval as the main independent variable. This was derived from asking the women the time preceding birth interval. Responses to this were stated in months. For the purpose of this study, this variable was categorised into 1 = less or equal to 2 years and 2 = more than 2 years.

#### 2.2.3. Control Variables

Twenty-one (21) control variables were considered for our study. These variables were made up of maternal factors, child factors, and contextual factors. The maternal factors were mother's age, mother's age at birth, husband/partner's age, mother's religion, mother's educational level, mother's employment status, father's employment status, sexual autonomy, contraceptive use, mother's experience of IPV, mother's exposure to mass media, and desire for pregnancy. The child factors were the sex of the child, size of the child at birth, birth order, the type of delivery, delivery assistance, place of delivery, and antenatal care. Place of residence, wealth quintile, and country were the contextual factors. These variables were not determined a priori; instead, based on parsimony, theoretical relevance, and practical significance with under-five mortality [15–18].

### 2.3. Statistical Analyses

Analysis for the study was done using Stata version 16.0. Univariate, bivariate, and multilevel and binary logistic regression analyses were conducted. Frequencies and proportions were first conducted, followed by a distribution of under-five mortality per the explanatory variables considered in this study, with their respective confidence intervals (CIs). After this, we carried out a multilevel logistic regression analysis to examine the factors associated with under-five mortality using five models. Model 0 showed the variance in under-five mortality attributed to the clustering of the primary sampling units (PSUs) without the explanatory variables. Model 1 contained the main independent variable (birth interval) and under-five mortality. Model 2 and 3 contained the child and maternal factors, respectively, and under-five mortality, while Model 4 contained the contextual factors. Model 5 contained all the independent and control variables. The Stata command “melogit” was used in fitting these models. We used Akaike's Information Criterion (AIC) tests for model comparison. Finally, we stratified our analysis by country to examine the heterogeneity in under-five mortality in all countries using binary logistic regression. Statistical significance was considered at a *p* value less than 0.05 and odds ratios at 95 percent confidence intervals were determined. This was followed by a multivariable binary logistic regression. Statistical significance was considered at a *p* value less than 0.05 and odds ratios at 95 percent confidence intervals were determined. All frequency distributions were weighted, while the survey command (SVY) in Stata was used to adjust for the complex sampling structure of the data in the regression analyses.

## 3. Results

### 3.1. Characteristics of Respondents and Under-Five Mortality Rate in West Africa

Table 2 shows the distribution of under-five mortality across birth interval and other characteristics of women. The highest under-five mortality rate was found among mothers with ≤2 years (108 deaths per 1000 live births), those in rural areas (85 deaths per 1000 live births), those with the poorest wealth quintile (95 deaths per 1000 live births), mothers aged <20 years (108 deaths per 1000 live births), mothers whose age at first childbirth were <20 years (84 deaths per 1000 live births), and women whose partners were 50+ years (94 deaths per 1000 live births). Muslim women (82 deaths per 1000 live births), women with no formal education (88 deaths per 1000 live births), employed women (77 deaths per 1000 live births), women whose partners were employed (75 deaths per 1000 live births), those who were not exposed to media (82 deaths per 1000 live births), and those whose pregnancies were intended (77 deaths per 1000 live births) had the highest under-five mortality rates. Disparities in under-five mortality rate were also found among the sex of children, size of child at birth, birth order, sexual autonomy, type of birth, place of delivery, delivery assistance, ANC attendance, mother's experience of IPV, and contraceptive use.

### 3.2. Association between Birth Interval and Under-Five Mortality

As shown in Table 3, the birth interval had a significant independent association with under-five mortality, with children born to mothers who had >2 years birth interval less likely to die before the age of five compared to mothers with ≤2 years birth interval [cOR = 0.56; CI = 0.51 − 0.62], and this persisted after controlling for the covariates [aOR = 0.55; CI = 0.50 − 0.61]. In terms of the covariates, children born to women aged 20-29 years [aOR = 0.74; CI = 0.60 − 0.90] were less likely to die before 5 years compared to those aged <20 years while children born to women whose partners were 50+ years were more likely to die compared to those whose partners were aged 15-19 [aOR = 1.23; CI = 1.03 − 1.47]. Children whose mothers were employed were more likely to die before age five compared to those born to unemployed mothers [aOR = 1.16; CI = 1.02 − 1.33]. Children whose mothers were exposed to mass media were more likely to die compared to those whose mothers were not exposed [aOR = 1.12; CI = 1.02 − 1.23]. Children whose mothers' current pregnancy was intended were more likely to die before age five compared to those whose mothers' current pregnancy was unintended. Women who were using contraceptives at the time of the survey were less likely to have experienced under-five mortality compared to those who were not using contraceptives [aOR = 0.70; CI = 0.62 − 0.79]. The odds of under-five mortality were lower among female children and 2-3 birth order children but higher among very small children, compared to male children, first birth order children, and very large children. Children born to mothers with the richest wealth quintile were less likely to die before age five compared to those born to the poorest mothers [aOR = 0.72; CI = 0.59 − 0.87]. Finally, compared to Burkina Faso, the death of children under five was lower in Benin, Gambia, Mali, and Togo but higher in Sierra Leone (Table 3).

The country-specific results showed that children born to mothers who had >2 years birth interval were less likely to die before the age of five compared to mothers with ≤2 years birth interval in all the eight countries (Table 4).

## 4. Discussion

The focus of this study was to assess the association between birth interval and under-five mortality in eight West African countries, using data from the DHS. From the study, the odds of children dying before age 5 was higher among children born to mothers who had more than a 2-year space between the previous birth compared to children born to mothers who had less or equal to 2 years interval. This confirms previous studies on the association between birth interval and under-five mortality [19] such as other low- and middle-income countries such as Kenya [20], South Africa [21], and Bangladesh [22]. A possible explanation for this is that mothers that waited for more than 18 months before having the next baby would have regained most body nutrients and blood loss during previous pregnancy and breastfeeding. It is also a common medical knowledge that the risk of obstetrics complications is higher in mothers that had a short birth interval than those with long birth interval.

Children from the richest households, those whose mothers age were 20-29, those whose mothers gave birth by age 20-29, and those whose mothers said their current pregnancy were mistimed and unwanted had a lower risk of under-five mortality. On the contrary, children whose fathers were 50 years and above, whose size at birth were smaller than average and very small, and those whose mothers had caesarean section have a higher risk of under-five mortality. These findings are consistent with previous reports [23–28]. From this study, it was seen that children of women who are employed were more likely to die before their 5^th^ birthday. This finding contradicts the finding of previous studies [27–29].

The study further established that under-five mortality varied by child sex, with female children having smaller odds of dying before their 5^th^ birthday, compared to male children. Several studies confirm this finding [8, 30–32]. Two explanations can be drawn from this finding. This explanation can be seen as biological and social. Biologically, male children who are susceptible to infection, more likely to be born premature, have a larger average body size and big head circumference which extend the time for mothers giving birth to male children [8, 31, 33]. Socially, gender discrimination in relation to feeding and medical care practices exists among male and female children, with the practices favoring females [8, 31, 33]. From this study, it was seen that children with a birth order of 2-3 were less likely to die before their 5^th^ birthday compared to children who are firstborns. This finding contradicts the findings of previous studies which state that the higher birth orders the higher the likelihood of under-five mortality [34]. The findings of the study also established that there is an association between contraceptive use and under-five mortality. The likelihood of under-five mortality reduced in children of mothers that embraced the use of contraception.

### 4.1. Strengths and Limitations

Our study's strength lies in the use of nationally representative datasets from eight countries in West Africa under the DHS program. This fortifies the generalizability of our study findings to the general population of young women. Moreover, the use of binary logistics regression and a large sample size ensured the reliability and replicability of the study to other populations. Notwithstanding the apparent strengths of our study, we caution that our findings be interpreted in the light of some limitations. Given that the dataset used employed cross-sectional designs, it limits the possibility of causal inferences primarily because of the snapshot nature of such design. The data used in this study is a retrospective self-reporting data, and as such, it may be biased by the mother's perception or feeling about pregnancy which might change over time.

## 5. Conclusion

We conclude that shorter birth intervals are associated with higher under-five mortality. Wealth quintile, mother's age, mother's age at first birth, partner's age, employment status, current pregnancy intention, sex of child, size of child at birth, birth order, type of birth, and contraceptive use also had associations with under-five mortality. Reproductive health interventions aimed at reducing under-five mortality should focus on lengthening birth intervals. Such interventions should be implemented taking into consideration the characteristics of women and their children.

## Figures and Tables

**Table 1:** Sample.

Country	Year	Weighted (*n*)	Weighted (%)	Under-five mortality rate (per 1000 live births)
Benin	2017/2018	7680	14.52	72
Burkina Faso	2010	9580	18.12	94
Cote D'Ivorie	2011-12	3489	6.60	72
Gambia	2013	4183	7.91	36
Mali	2018	5463	10.33	69
Nigeria	2018	13234	25.03	96
Sierra Leone	2019	5374	10.16	100
Togo	2013-14	3873	7.33	57
Total	—	52877	100	74

**Table 2:** Characteristics of respondents and under-five mortality rate in West Africa.

Variables	Weighted *N*	Weighted %	Under-five mortality
Birth interval			
≤2 years	7681	14.5	108 [96-120]
>2 years	45196	85.5	68 [63-73]
Place of residence			
Urban	18096	34.2	56 [50-61]
Rural	34780	65.8	85 [77-94]
Wealth quintile			
Poorest	10700	20.2	95 [81-106]
Poorer	11067	20.9	93 [80-105]
Middle	11009	20.8	75 [66-84]
Richer	10541	19.9	71 [61-80]
Richest	9560	18.1	40 [33-47]
Mother's age			
<20 years	2705	5.1	108 [80-136]
20-29 years	24461	46.3	67 [60-74]
30-49 years	25711	48.6	77 [72-82]
Mother's age at first birth			
<20 years	30572	57.8	84 [78-90]
20-29 years	21136	40.0	63 [55-70]
30-49 years	1169	2.2	47 [31-62]
Partner's age			
<30 years	7746	14.7	78 [60-95]
30-39 years	20743	39.2	62 [57-68]
40-49 years	15802	29.9	74 [66-82]
50+ years	8586	16.2	94 [85-103]
Religion			
Christian	13881	26.2	62 [55-69]
Islam	31390	59.4	82 [75-89]
Others	7606	14.4	69 [57-80]
Educational level			
No education	31565	59.7	88 [80-96]
Primary	8895	16.8	69 [60-78]
Secondary	10423	19.7	47 [40-53]
Higher	1993	3.8	39 [30-48]
Employment status			
Unemployed	9150	17.3	62 [51-73]
Employed	43727	82.7	77 [72-82]
Partner's employment status			
Unemployed	1092	2.1	44 [27-61]
Employed	51785	97.9	75 [71-80]
Exposure to mass media			
Not exposed	17382	32.9	82 [75-90]
Exposed	35.495	67.1	71 [66-76]
Current pregnancy intended			
Then	39061	743.8	79 [73-85]
Later	3130	5.9	66 [53-79]
No more	10730	20.3	63 [54-72]
Sex of child			
Male	26958	51.0	81 [75-87]
Female	25919	49.0	68 [62-74]
Size of child at birth			
Very large	6538	12.4	63 [51-76]
Larger than average	12732	24.1	66 [56-75]
Average	26054	49.3	75 [69-81]
Smaller than average	5626	10.6	94 [80-109]
Very small	1927	3.6	114 [91-138]
Birth order			
First	8414	15.9	71 [62-79]
2-3	18353	34.7	53 [48-60]
4 or more	26110	49.4	88 [82-95]
Sexual autonomy			
No	11263	21.3	82 [69-95]
Yes	41614	78.7	72 [68-77]
Type of birth			
Virginal birth	50896	96.3	74 [69-79]
Caesarean birth	1981	3.7	90 [65-114]
Place of delivery			
Home	17035	32.2	103 [92-113]
Health facility	35842	67.8	61 [56-66]
Delivery assistance			
TBA/others	12335	23.3	107 [94-121]
SBA/health professionals	40542	76.7	65 [59-70]
ANC attendance			
No	22487	42.5	89 [82-96]
Yes	30390	57.5	65 [59-70]
Mother experience IPV			
No	43965	83.1	74 [69-79]
Yes	8912	16.9	77 [63-91]
Contraceptive use			
No	42958	81.2	84 [76-91]
Yes	9919	18.8	42 [35-49]

**Table 3:** Multilevel regression analysis of the association between under-five mortality in West Africa and sociodemographic and behavioral characteristics.

Variables	Model 0	Model 1 cOR (95% CI)	Model 2 aOR (95% CI)	Model 3 aOR (95% CI)	Model 4 aOR (95% CI)	Model 5 aOR (95% CI)
Birth interval						
≤2 years		Ref				Ref
>2 years		0.56^∗∗∗^ (0.51-0.62)				0.55^∗∗∗^ (0.50-0.61)
Mother's age						
<20 years			Ref			Ref
20-29 years			0.73^∗∗∗^ (0.61-0.88)			0.74^∗∗^ (0.60-0.90)
30-49 years			0.96 (0.78-1.18)			0.88 (0.69-1.13)
Mother's age at first birth						
<20 years			Ref			Ref
20-29 years			0.86^∗∗∗^ (0.78-0.94)			0.93 (0.84-1.02)
30-49 years			0.74 (0.54-1.01)			0.78 (0.57-1.09)
Partner's age						
<30 years			Ref			Ref
30-39 years			0.88 (0.77-1.01)			0.88 (0.76-1.02)
40-49 years			1.01 (0.86-1.18)			0.99 (0.84-1.17)
50+ years			1.25^∗^ (1.05-1.48)			1.23^∗^ (1.03-1.47)
Religion						
Christian			Ref			Ref
Islam			1.05 (0.95-1.17)			1.09 (0.97-1.22)
Others			0.92 (0.80-1.06)			1.04 (0.86-1.25)
Educational level						
No education			Ref			Ref
Primary			0.97 (0.87-1.09)			1.02 (0.90-1.15)
Secondary			0.85^∗^ (0.75-0.96)			0.88 (0.77-1.02)
Higher			0.76 (0.57-1.00)			0.81 (0.60-1.10)
Employment status						
Unemployed			Ref			Ref
Employed			1.39^∗∗∗^ (1.23-1.57)			1.16^∗^ (1.02-1.33)
Partner's employment status						
Unemployed			Ref			Ref
Employed			1.20 (0.86-1.66)			1.28 (0.92-1.77)
Sexual autonomy						
No			Ref			Ref
Yes			1.05 (0.95-1.16)			0.99 (0.89-1.11)
Exposure to mass media						
Not exposed			Ref			Ref
Exposed			0.95 (0.88-1.04)			1.12^∗^ (1.02-1.23)
Current pregnancy intended						
Then			Ref			Ref
Later			0.81^∗^ (0.68-0.98)			0.75^∗∗^ (0.62-0.90)
No more			0.75^∗∗∗^ (0.66-0.84)			0.70^∗∗∗^ (0.62-0.79)
Mother experience IPV						
No			Ref			Ref
Yes			1.10 (0.99-1.22)			1.08 (0.97-1.21)
Contraceptive use						
No			Ref			Ref
Yes			0.69^∗∗∗^ (0.61-0.78)			0.70^∗∗∗^ (0.62-0.79)
Sex of child						
Male				Ref		Ref
Female				0.82^∗∗∗^ (0.76-0.89)		0.83^∗∗∗^ (0.76-0.90)
Size of child at birth						
Very large				Ref		Ref
Larger than average				0.92 (0.79-1.06)		0.89 (0.77-1.04)
Average				1.03 (0.90-1.17)		0.96 (0.84-1.10)
Smaller than average				1.29^∗∗^ (1.09-1.52)		1.24^∗^ (1.05-1.47)
Very small				2.01^∗∗∗^ (1.65-2.44)		2.07^∗∗∗^ (1.70-2.53)
Birth order						
First				Ref		
2-3				0.79^∗∗∗^ (0.69-0.90)		0.72^∗∗∗^ (0.62-0.84)
4 or more				1.22^∗∗∗^ (1.09-1.38)		1.02 (0.86-1.23)
Type of birth						
Virginal birth				Ref		Ref
Caesarean birth				1.81^∗∗∗^ (1.50-2.18)		
ANC attendance						
No				Ref		Ref
Yes				0.90^∗^ (0.83-0.98)		0.96 (0.87-1.05)
Place of delivery						
Home				Ref		Ref
Health facility				0.77^∗∗∗^ (0.68-0.87)		0.97 (0.85-1.11)
Delivery assistance						
TBA/others				Ref		Ref
SBA/health professionals				0.99 (0.87-1.12)		0.91 (0.79-1.03)
Place of residence						
Urban					Ref	Ref
Rural					1.04 (0.93-1.16)	1.01 (0.90-1.14)
Wealth quintile						
Poorest					Ref	Ref
Poorer					0.97 (0.87-1.09)	1.04 (0.92-1.17)
Middle					0.86^∗^ (0.76-0.97)	0.96 (0.85-1.09)
Richer					0.83^∗∗^ (0.73-0.95)	1.00 (0.87-1.16)
Richest					0.53^∗∗∗^ (0.45-0.63)	0.72^∗∗∗^ (0.59-0.87)
Country						
Burkina Faso					Ref	Ref
Benin					0.77^∗∗∗^ (0.66-0.89)	0.81^∗^ (0.69-0.96)
Cote d'Ivorie					1.03 (0.86-1.22)	1.07 (0.89-1.29)
Gambia					0.44^∗∗∗^ (0.36-0.55)	0.39^∗∗∗^ (0.30-0.49)
Mali					0.82^∗^ (0.70-0.55)	0.77^∗∗^ (0.64-0.92)
Nigeria					1.16^∗^ (1.03-1.30)	1.14 (0.99-1.32)
Sierra Leone					1.13 (0.97-1.31)	1.30^∗∗^ (1.10-1.55)
Togo					0.70^∗∗∗^ (0.58-0.85)	0.78^∗^ (0.61-1.00)
Random effect result						
PSU variance (95% CI)	0.07 (0.04-0.13)	0.07 (0.04-0.13)	0.06 (0.03-0.12)	0.06 (0.03-0.12)	0.05 (0.02-1.00)	0.04 (0.02-0.10)
ICC	0.0211678	0.0207558	0.0180652	0.0185152	0.0139181	0.0132386
LR test	15.92 (*X*^2^ = 0.0000)	15.37 (*X*^2^ = 0.0000)	12.03 (*X*^2^ = 0.0003)	12.46 (*X*^2^ = 0.0002)	7.51 (*X*^2^ = 0.0031)	6.74 (*X*^2^ = 0.0047)
Wald chi-square		131.30^∗∗∗^	233.56^∗∗∗^	282.90^∗∗∗^	209.09^∗∗∗^	721.41^∗∗∗^
Model fitness						
Log-likelihood	-10031.127	-99714453	-9909.8115	-9893.1726	-9915.005	-9661.2923
AIC	20066.25	19948.89	19863.62	19812.35	19858.01	19414.58
N	52877	52877	52877	52877	52877	52877

^∗^ *p* < 0.05, ^∗∗^*p* < 0.01, ^∗∗∗^*p* < 0.001.

**Table 4:** Binary regression analysis of the association birth interval and under-five mortality in West Africa disaggregated by county.

Country	Model 1 cOR (95% CI)	Model 2 aOR (95% CI)
Benin	0.51^∗∗∗^ (0.39-0.65)	0.52^∗∗∗^ (0.40-0.67)
Burkina Faso	0.68 (0.50-0.91)	0.64^∗∗^ (0.47-0.89)
Cote d'Ivorie	0.53^∗∗^ (0.36-0.76)	0.55^∗∗^ (0.37-0.82)
Gambia	0.58^∗∗^ (0.35-0.96)	0.47^∗∗^ (0.27-0.82)
Mali	0.63^∗∗∗^ (0.46-0.86)	0.63^∗∗^ (0.45-0.88)
Nigeria	0.67^∗∗∗^ (0.56-0.79)	0.65^∗∗∗^ (0.54-0.76)
Sierra Leone	0.39^∗∗∗^ (0.30-0.51)	0.38^∗∗∗^ (0.29-0.51)
Togo	0.44^∗∗∗^ (0.29-0.67)	0.37^∗∗∗^ (0.24-0.58)

## Data Availability

Data for this study were sourced from Demographic and Health surveys (DHS) and available here: http://dhsprogram.com/data/available-datasets.cfm.

## Abbreviations

AOR: Adjusted odds ratio
CI: Confidence interval
DHS: Demographic and health survey
LMIC: Low- and middle-income countries
SDGs: Sustainable development goal
SSA: Sub-Saharan Africa.
